# Local Anæsthesia and the Dentists

**Published:** 1891-07

**Authors:** Dwight M. Clapp

**Affiliations:** Boston


### LOCAL ANÆSTHESIA AND THE DENTISTS.[1]

BY DWIGHT M. CLAPP, D.M.D., BOSTON.

We shall publish in our next number a study of the legal aspect of this subject, but just before going to press we received a pamphlet from our *confrère*, M. Bouchard, dentist, at Lille, giving

[1] Translated from *L'Odontologie*, January, 1891.

an account of a fatal accident that occurred in his office. The subject is of so great interest that we give the following extract immediate publication :

"Miss X. called on the 7th of August to make an appointment for the following day, Friday, at seven o'clock, A.M. On that day I proceeded to extract two superior teeth after having made two external and one internal subcutaneous injections of a solution (cocaine) of one to one hundred,—*au centième ;* the teeth were very loose and the success was complete. After several minutes my patient expressed a wish to have another tooth in the left inferior jaw, and equally as loose as the others, extracted. I injected the rest of the cocaine in my syringe and after waiting a few minutes extracted the tooth. The wound did not bleed. I gave the patient a little warm water and a little black coffee to induce the flow of blood. Nothing appeared to indicate an accident; the patient seemed to be all right, although somewhat fatigued. I left her in the care of her uncle while I went for more coffee.

"On my return the young lady had fainted. She was placed on the floor, her clothing loosened, dashes of cold water, friction,—in short, everything was done that the situation indicated. The physician, hastily summoned, continued the same treatment, and, in addition, gave nitrate of amyl, oxygen, and subcutaneous injections of ether ; but the patient succumbed after a half-hour of unsuccessful treatment.

"The physician who made the death certificate, after describing the fainting of the patient, concluded that death was caused by cocaine, as that salt had been employed.

"Was cocaine the cause of death ? I will prove the contrary.

"When the honorable doctor incriminated cocaine he was ignorant, as I was myself, at the time, that a large knotted cord, called *corde à lessive*, was wound four times around the body of the girl, compressing the thorax. This cord was drawn so tightly that its presence was not suspected and could not be cut without injury to the skin.

"This explains why the attempts at artificial respiration were unsuccessful."

